# The brain–heart interaction in epilepsy: implications for diagnosis, therapy, and SUDEP prevention

**DOI:** 10.1002/acn3.51382

**Published:** 2021-05-28

**Authors:** Giorgio Costagliola, Alessandro Orsini, Monica Coll, Ramon Brugada, Pasquale Parisi, Pasquale Striano

**Affiliations:** ^1^ Pediatric Clinic Santa Chiara’s University Hospital Azienda Ospedaliero‐Universitaria Pisana Pisa Italy; ^2^ Cardiovascular Genetics Center Institut d'Investigació Biomèdica de Girona (IDIBGI) Girona Spain; ^3^ Cardiovascular Genetics Center Institut d'Investigació Biomèdica de Girona (IDIBGI) Girona Spain; ^4^ Medical Science Department School of Medicine University of Girona Girona Spain; ^5^ Centro de Investigación Biomédica en Red de Enfermedades Cardiovasculares (CIBERCV) Madrid Spain; ^6^ Cardiology Service Hospital Josep Trueta Girona Spain; ^7^ Chair of Pediatrics, NESMOS Department Faculty of Medicine and Psychology Sapienza University Sant' Andrea Hospital Rome Italy; ^8^ IRCCS Istituto Giannina Gaslini Genova Italy; ^9^ Department of Neuroscience, Rehabilitation, Ophthalmology, Genetics, Maternal and Child Health University of Genova Genova Italy

## Abstract

The influence of the central nervous system and autonomic system on cardiac activity is being intensively studied, as it contributes to the high rate of cardiologic comorbidities observed in people with epilepsy. Indeed, neuroanatomic connections between the brain and the heart provide links that allow cardiac arrhythmias to occur in response to brain activation, have been shown to produce arrhythmia both experimentally and clinically. Moreover, seizures may induce a variety of transient cardiac effects, which include changes in heart rate, heart rate variability, arrhythmias, asystole, and other ECG abnormalities, and can trigger the development of Takotsubo syndrome. People with epilepsy are at a higher risk of death than the general population, and sudden unexpected death in epilepsy (SUDEP) is the most important direct epilepsy‐related cause of death. Although the cause of SUDEP is still unknown, cardiac abnormalities during and between seizures could play a significant role in its pathogenesis, as highlighted by studies on animal models of SUDEP and registration of SUDEP events. Recently, genetic mutations in genes co‐expressed in the heart and brain, which may result in epilepsy and cardiac comorbidity/increased risk for SUDEP, have been described. Recognition and a better understanding of brain–heart interactions, together with new advances in sequencing techniques, may provide new insights into future novel therapies and help in the prevention of cardiac dysfunction and sudden death in epileptic individuals.

## Introduction

Anatomic and functional connections between the heart and brain have substantial clinical and research implications, and there is increasing evidence on the role of the nervous system in modulating cardiac functioning. The influence on cardiologic function has a relevant impact on the clinical course and prognosis of individuals with epilepsy, being involved in the pathogenesis of epilepsy‐associated arrhythmias and sudden unexpected death in epilepsy (SUDEP).

Epilepsy is associated with a higher risk of death compared to the general population, with SUDEP being the most important direct epilepsy‐related cause of premature death.^1^ It is defined as the sudden, unexpected death in people with epilepsy independently of the evidence of a seizure when trauma, drowning, and status epilepticus have been excluded, and postmortem examination does not identify a cause of death.^2^ SUDEP occurs in about 1.4/1000 patients/year,^3^ with a peak of incidence in adolescents and young adults (under 45 years).^3^ The frequency is higher in individuals with refractory epilepsy, generalized tonic‐clonic seizures (GTCS), and patients with developmental and epileptic encephalopathy, with a higher incidence in Dravet syndrome (DS).^4^

The etiology and the pathogenic mechanisms leading to SUDEP are still unknown, as there are only a few recorded episodes of SUDEP; however, it occurs more frequently during the sleeping hours and in the postictal phase.^1^ Although different mechanisms, including respiratory dysfunction and post‐ictal brain depression, can contribute to the pathogenesis of SUDEP, there is a particular interest in the role of cardiac abnormalities during and between seizures, deriving from altered autonomic control of cardiac activity.^1^

Current knowledge does not allow predicting the occurrence of SUDEP, as no specific clinical, ECG, EEG, imaging, or laboratory biomarkers have been identified.^5^ Therefore, specific prevention strategies are lacking, and optimal seizure control is the most widely accepted protective factor. Nevertheless, the recent expansion of the use of next‐generation sequencing (NGS) and whole‐exome sequencing (WES) allowed the identification of mutations in genes co‐expressed in the heart and brain which may result in epilepsy and cardiac comorbidity/increased risk for SUDEP.^6^ Interest in the complex interplay between the brain and the cardiovascular system in epileptic individuals is not limited to SUDEP. Indeed, seizures may induce a variety of transient cardiac effects during the peri‐ictal phase, which includes the elevation of blood pressure (BP), changes in heart rate (HR), arrhythmias, and other ECG abnormalities, and epileptic individuals have a higher prevalence of cardiac comorbidities compared to the general population.^7^

We review the main anatomic and physiological connections between the nervous and the cardiovascular system, with a particular focus on epilepsy and SUDEP. Moreover, we analyze the main perspectives in this complicated field to guide further research on SUDEP prevention.

## The Brain**–**Heart Connections

**FIGURE 1:**
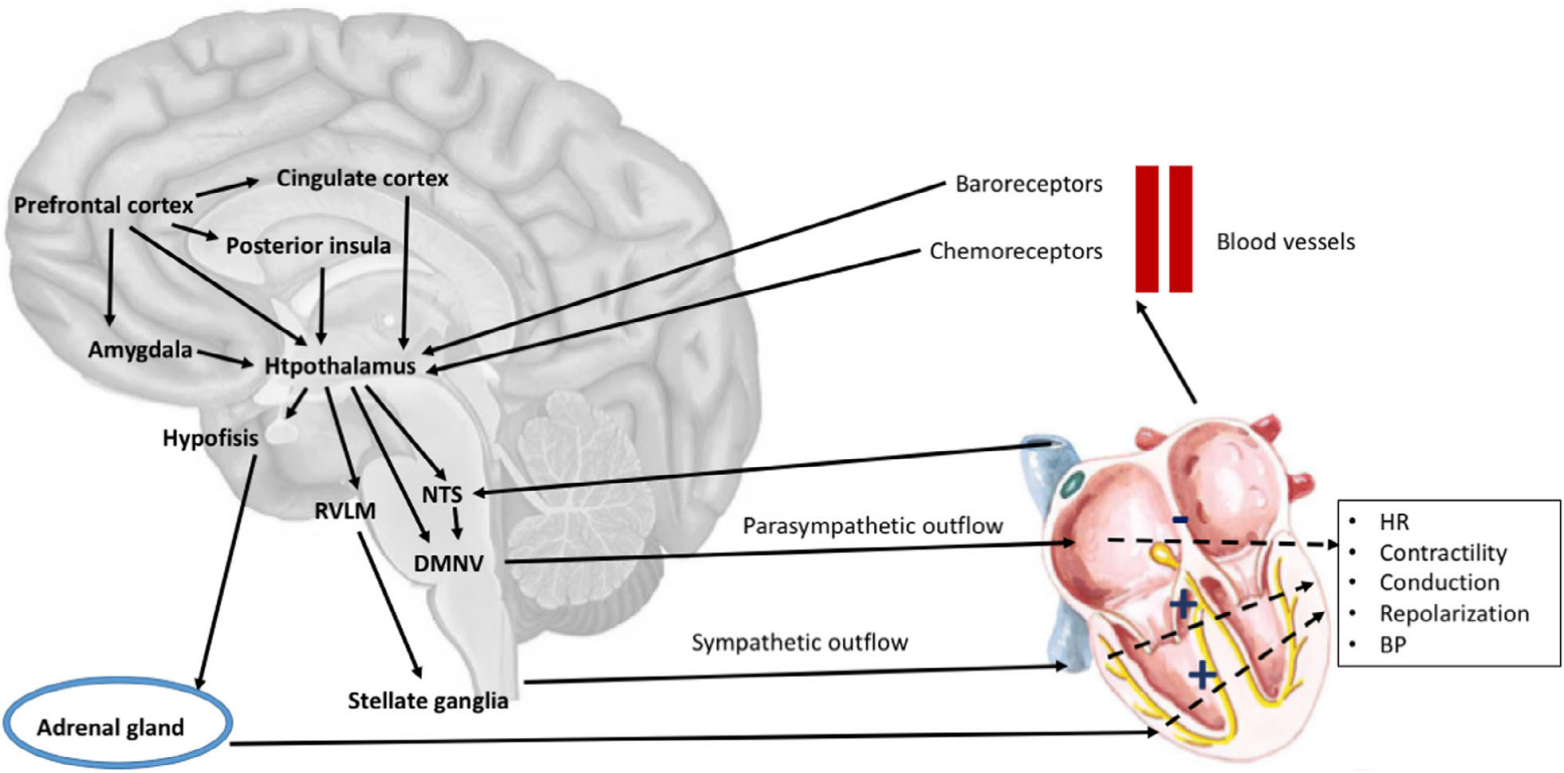
Overview of the main brain–heart connections. The figure summarizes, in a simplified modality, the most relevant anatomic, and functional connections between the brain and the cardiovascular system. DMNV, dorsal motor nucleus of the vagus; NTS, nucleus tractus solitaries, RVLM, rostral ventrolateral medulla.

Cortical areas and subcortical structures are related to cardiac function through their influence on the autonomic system, which represents the final effector that modulates cardiac activity.^8^ Moreover, feedback from the cardiovascular system can influence the autonomic outflow through the activation of neuro‐cardiac reflexes^8^, ^9^ (Fig. 1). Accordingly, results from animal models suggest that altered neuro‐cardiac connections could represent a risk factor for SUDEP.^10^

### The autonomic system and the heart

The regulation of cardiac electrical and mechanical activity from sympathetic and parasympathetic innervation depends on the influence of different ion channels expressed on the cardiomyocyte surface, regulating the cellular fluxes of calcium, sodium, and potassium.^11^ Through their modulation, the sympathetic system enhances cardiac conduction, HR, repolarization, contractility, and relaxation, while the parasympathetic system mediates the opposite actions.^12^ As increased sympathetic activity is responsible for a reduced arrhythmic threshold,^9^, ^12^ genetic predisposition to an increased response to autonomic innervation, evidenced in different disorders of ion channels (i.e., long QT syndrome [LQTS], Brugada syndrome, catecholaminergic polymorphic ventricular tachycardia), is associated with a high risk of arrhythmias and sudden cardiac death (SCD).^6^, ^13^

The brainstem has a central role in cardiac innervation, hosting the nuclei of parasympathetic nerves (the dorsal motor nucleus of the vagus, *nucleus ambiguus*), implicated in the innervation of the heart and in mediating the autonomic cardiac reflexes. Moreover, sympathetic innervation originates from the rostral ventrolateral medulla (RVLM), finding the postsynaptic neurons in the stellate ganglia.^12^

### Autonomic modulation

The hypothalamus plays a pivotal role in modulating autonomic activity and adrenal release of catecholamines.^14^, ^15^ Moreover, the suprachiasmatic nucleus is a regulator of the physiological circadian clock, and may directly affect the circadian rhythm of BP, HR, heart rate variability (HRV), and susceptibility to arrhythmias. In this regard, some authors suggested that circadian variations in cardiac and respiratory activity could contribute to the nocturnal prevalence of SUDEP.^16^

The influence of cortical functions on cardiac activity has been demonstrated in both clinical studies and animal models. Stimulation of different cortical areas can induce ECG changes, arrhythmias, or alter BP,^9^ with the posterior insula, the cingulate cortex, the prefrontal cortex, and the amygdala having the most prominent role in controlling autonomic function.^8^, ^9^ As components of the limbic system, these areas represent the point of connection between the emotional state, mental stress, and cardiovascular response.^17^

The amygdala has functional connections with the hypothalamus and structures of the brainstem, including RVLM and *nucleus tractus solitarius*, and thus influences the autonomic outflow and causes changes in cardiac activity and BP.^18^ Additionally, studies investigating the stimulation of the insula evidenced the role of the posterior insula in increasing sympathetic outflow.^8^

Apart from epilepsy, the role of the cortex in modulating cardiac function is confirmed by the cardiac alterations in different neurological disorders with cortical involvement. Indeed, in patients with ischemic and hemorrhagic stroke and subarachnoid hemorrhage, higher sympathetic activity is observed, and patients show an increased risk for new‐onset arrhythmias, EKG alterations, or myocardial injury.^8^

## Epilepsy and Cardiac Comorbidities

**FIGURE 2:**
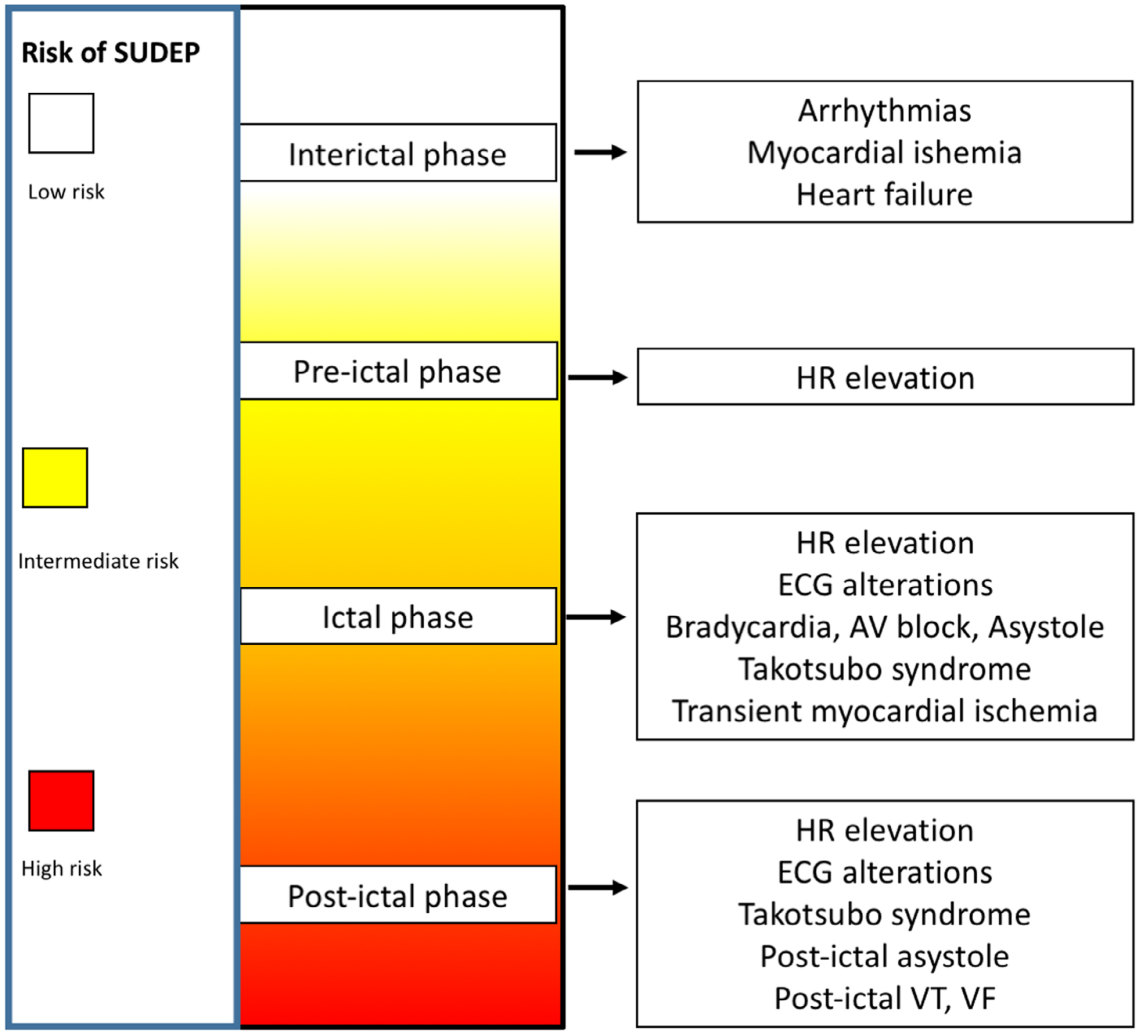
Cardiovascular involvement in epilepsy. The figure summarizes cardiac involvement in epileptic individuals, with a focus on the different clinical manifestations in the interictal period, pre‐ictal, ictal, and post‐ictal phase. HR, heart rate; TTS, Takotsubo syndrome; VF, ventricular fibrillation; VT, ventricular tachycardia.

People with epilepsy show a wide range of cardiologic manifestations, mainly triggered by seizures. Seizures are associated with variations of the HRV, HR, and BP, with an increased risk of developing arrhythmias, and can be implicated in triggering Takotsubo syndrome (TTS). Moreover, during seizures patients can develop transient myocardial ischemia,^7^ contributing to lowering the threshold for arrhythmias (Fig. 2), and epileptic individuals are also more likely to present structural and functional cardiac abnormalities. This increased prevalence of structural alterations could be the result of common genetic predisposing factors, as well as the consequence of sedentary lifestyle, overweight, and the use of antiseizure medications (ASMs) altering lipid metabolisms, such as carbamazepine and phenytoin.^7^ Different authors suggest that seizure activity could cause repetitive myocardial injury with a catecholamine‐mediated mechanism, leading to the concept of the “epileptic heart,” which is featured by chronic heart and coronary damage, resulting in myocardial fibrosis, accelerated atherosclerosis, systolic and diastolic dysfunction, and arrhythmias.^19^, ^20^ Concerning mechanical dysfunction, recent studies demonstrated a reduced systolic and diastolic function in individuals with epilepsy, without evidence of a correlation between the echocardiographic findings and the severity of epilepsy.^21^, ^22^ Cardiac fibrosis and vascular dysfunction contribute to altering cardiac conduction and repolarization, thus generating a substrate for the development of arrhythmias.^23^ Finally, as a result of the structural, functional, and electrical anomalies, a higher incidence of myocardial infarction (4.83‐fold increase) in epileptic individuals compared to the general population has been demonstrated.^24^

### Heart rate variability

HRV and HR trend have been extensively studied as a potential predictor of seizure severity and for the potential implications in the pathogenesis of SUDEP.^25^ Indeed, HRV reflects the balance of the autonomic outflow, its decrease being associated with sympathetic dominance.

In the peri‐ictal period, a significant decrease in HRV is observed, particularly in people with temporal lobe epilepsy (TLE) and GTCS.^25^ Enhanced sympathetic activity, together with the suppression of parasympathetic outflow, is also responsible for HRV changes in the post‐ictal phase.^26^ Additionally, several studies analyzed interictal HRV to identify epileptic individuals with increased risk for SUDEP. Although people with drug‐resistant epilepsy show low interictal HRV,^27^, ^28^ which can potentially contribute to the pathogenesis of SUDEP, its predictive role is still undetermined.^29^ A recent paper analyzed HR and HRV at rest, during, and after hyperventilation performed during the patient's last EEG recording before SUDEP. The authors showed that these patients have an abnormal cardiac autonomic response to sympathetic stimulation through hyperventilation and suggested that an index reflecting the change in HR upon hyperventilation could be used to select patients at risk of SUDEP.^30^

The effects of sympathetic prevalence are evidenced also on HR, with up to 80% of the patients showing a continuous increase of HR from pre‐ictal to ictal phase,^31^ while the finding of a reduced HR is less common.^31^ A recent meta‐analysis evidenced that HR alterations are more common in people with TLE and that a pre‐ictal increase in HR is 2‐fold more frequent in the adult population, while its decrease is reported almost exclusively in children.^32^ There is evidence of changes in HR also during the post‐ictal phase.

Arbune et al. showed that an elevated mean post‐ictal HR was associated with higher duration and severity of the seizure event.^26^ Moreover, several ECG findings, including prolonged QTc interval, ST abnormalities, and altered repolarization have been reported during the peri‐ictal period.^7^

### Epilepsy and arrhythmias

Peri‐ictal arrhythmias can be evidenced as the result of the autonomic imbalance deriving from seizure activity. During the ictal phase, the most commonly diagnosed arrhythmia is sinus tachycardia, while the most frequent clinically relevant arrhythmia is asystole,^33^ which is usually self‐limiting and has a higher incidence in individuals with TLE.^34^ The pathogenesis of asystole, as well as sinus bradycardia and atrioventricular (AV) block, can be ascribed to different mechanisms, including the stimulation of the limbic cortex activating parasympathetic outflow, and the sympathetic activation followed by vagal cardioinhibitory reflex.^7^ A systematic review by van der Lende et al found the occurrence of ictal asystole in 0.3% of people with refractory epilepsy.^33^ During the post‐ictal period, patients can occasionally develop arrhythmias including asystole, bradycardia, and AV block, but atrial flutter, atrial fibrillation (AF), ventricular tachycardia (VT), or ventricular fibrillation (VF) have been also reported.^33^ Post‐ictal arrhythmias have been reported after GTCS and, unlike ictal arrhythmias, show frequent association with near‐SUDEP,^33^ needing medical resuscitation.

Overall, epileptic individuals show a higher prevalence of arrhythmias compared to the general population. This association partly depends on a shared genetic susceptibility between epilepsy and arrhythmia, on which ion channel disorders (channelopathies) play a major role (Table 1). Moreover, the epileptic activity can induce cardiac electrophysiological alterations, leading to acquired channelopathies,^11^ and the use of antiepileptic drugs targeting sodium channels can be associated with AV conduction blocks, QT prolongation, or other ECG alterations.^7^

**Table 1:** Main channelopathies associated with epilepsy and arrhythmias.

Gene	Protein	Effect on brain	Effect on heart
KCNQ1	Potassium channel Kv7.1	Epilepsy	Long QT syndrome
KCNQ2	Potassium channel Kv7.2	Benign neonatal epilepsy; epileptic encephalopathy	Long QT syndrome
KCNH2	Potassium channel Kv11.1	Epilepsy	Long QT syndrome, Short QT syndrome
KCNJ2	Potassium channel Kir2.1	Epilepsy, autism spectrum disorder	Short QT syndrome, Long QT syndrome
KCNA1	Potassium channel Kv1.1	Epilepsy, ataxia	Atrial fibrillation, AV blocks
SCN1A	Sodium channel Nav1.1	Dravet syndrome	Likely increased risk of peri‐ictal arrhythmia
SCN2A	Sodium channel Nav1.2	Benign neonatal epilepsy; epileptic encephalopathy	Likely increased risk of arrhythmia
SCN5A	Sodium channel Nav1.5	Epilepsy	Long QT syndrome, Brugada syndrome
SCN8A	Sodium channel Nav1.6	Epileptic encephalopathy, movement disorders	Ventricular arrhythmias
SCN10A	Sodium channel Nav1.8	Epileptic encephalopathy	Long QT syndrome, Brugada syndrome
HCN1	Hyperpolarization‐activated cationic channel HCN1	Epileptic encephalopathy	Sick sinus syndrome
HCN4	Hyperpolarization‐activated cationic channel HCN4	Benign myoclonic epilepsy in infancy, generalized epilepsy	Sick sinus syndrome
CACNA1C	L‐type calcium channel Cav1.2 alpha 1	Epileptic encephalopathy, Timothy syndrome	Long QT syndrome, Short QT syndrome, Brugada syndrome, idiopathic VF
CACNA2D1	L‐type calcium channel Cav1.2 alpha 2‐delta 1	Epilepsy	Brugada syndrome, Short QT syndrome
RYR2	Ryanodine receptor 2 (intracellular calcium channel)	Epilepsy	CPVT

References: Devisnki et al. (2016),^1^ Chalhal et al. (2020),^6^ Coll et al. (2015),^35^ Thom et al. (2018),^36^ Bagnall et al. (2017),^37^ Goldman et al. (2016),^38^ Glasscock et al. (2015),^39^ Trosclair et al. (2020),^40^ Trosclair et al. (2021),^41^ Glasscock et al. (2019),^42^ Frasier et al. (2016).^43^

AV, atrioventricular; CPVT, Catecholaminergic polymorphic ventricular tachycardia; VF, Ventricular fibrillation.

### Takotsubo syndrome

TTS represents a paradigmatic condition showing the link between mental stress, cortical activation, and cardiac disease. It is characterized by a myocardial infarction‐like clinical picture associated with acute systolic apical left ventricular dysfunction, triggered by physical or emotional stress.^44^ Although initial misdiagnosis with acute coronary syndromes is common and ECG and laboratory findings are non‐specific, the absence of coronary disease at angiography, and the evidence of apical ballooning in left ventriculography, and circumferential abnormalities of wall motion at echocardiography significantly help in the diagnostic process.^45^ Histological findings, including enlarged myocytes, cytoskeletal rearrangement, and damage of contractile proteins, support the pathogenic role of catecholamine overload in TTS.^46^

Neurologic disorders (stroke, subarachnoid hemorrhage, and seizures) are the most frequent medical conditions associated with TTS,^47^ which can be detected immediately after the seizure episode or in the following hours, often in the absence of the typical clinical signs (chest pain, dyspnea).^48^

Seizure‐associated TTS is rare, although its prevalence is likely underestimated. A recent study showed a prevalence of 0.1% of TTS in patients hospitalized for seizures (5‐fold higher compared to the general population), with a higher frequency in patients with status epilepticus and GTCS.^49^ Additionally, female sex, acute infections, coronary atherosclerosis, and related risk factors (obesity, dyslipidemia) were associated with the development of TTS,^49^ while an association between a specific etiology of seizures and TTS has not been demonstrated. Currently, there is no evidence allowing to confirm the hypothesis of a relationship between seizure‐associated TTS and SUDEP.^50^

## Cardiac Changes in SUDEP

Previous research identified cardio‐autonomic and respiratory dysfunction as a frequent accompaniment in human and animal models of SUDEP. The role of cardiac dysfunction has been studied using animal models, autopsy, and through the direct registration of SUDEP events with cardiorespiratory and EEG monitoring.^1^, ^38^ The cardiac anomalies most often identified in registered events are arrhythmias such as tachycardia, bradycardia, T‐wave disturbances, ST elevation, asystole, AV block, AF, and VF, the latter often due to the lengthening or shortening of the QT interval. In autopsies, structural alterations of the myocardium such as interstitial fibrosis, myocyte hypertrophy or vacuolization, arteriolar wall thickening, and myocardial contraction band or transient left ventricular dysfunction have also been described.^51^

*Kcna1*−/− null mice represent an interesting model of SUDEP, in which seizures evoke respiratory dysfunction followed by cardiac abnormalities.^52^ Concerning SUDEP in DS, most studies suggest that respiratory dysfunction accelerates cardiac failure.^53^ *Scn1a*ΔE26 mice have spontaneous seizures and die prematurely, manifest hypoventilation under baseline conditions, and have a reduced CO2/H+ ventilatory response, showing an increased basal systolic arterial pressure and HR.^53^ In other animal models of DS (*Scn1a*R1407X/+ mutation), seizures are responsible for the development of central apnea, followed by bradycardia, and death.^54^

Other studies have shown that mice carrying de novo mutations in *SCN8A* show increased sodium current density and die prematurely in a dose‐dependent manner, while a recent study on mice with *SCN8A* mutation identified central apnea as the first pathogenic event leading to asystole and SUDEP.^11^, ^52^, ^55^ Also, baboons and sheep have been studied as animal models of SUDEP. In a recent study, epileptic baboons in a captive pedigree exhibited QT prolongation, and possibly reduced HRV compared to their asymptomatic relatives, thus identifying two variables that are potential biomarkers for SUDEP also in humans.^56^ Moreover, some authors developed a model of SUDEP using sheep in which GTCS status epilepticus was induced, and demonstrated that respiratory disease was the first process in developing SUDEP. Comparing the sudden death and long‐lived groups, the authors did not find any significant differences in the arrhythmias produced or malignant rhythm alteration, while marked differences in ventilation were found.^57^, ^58^, ^59^ However, although different studies evidenced that respiratory dysfunction could be the first alteration in SUDEP and precede cardiac involvement,^54^, ^55^ data are still conflicting, and further research is needed to clarify the physiopathology of SUDEP.

## Diagnostic and Therapeutic Implications

### Genetic analysis

When the scene investigation, autopsy, or toxicological study do not reveal a probable or definite cause of death, post‐mortem genetic analysis can reveal a variant that could cause sudden death.^36^ While a molecular autopsy is a well‐recognized approach in SCD, its role in SUDEP remains less well defined. Indeed, mutations in ion channel genes play a major part in the pathogenesis of several epilepsy syndromes (e.g., *SCN1A* in DS).^60^ Voltage‐gated channels play an essential role in neuronal excitability and it is not surprising that mutations associated with epilepsy may affect cardiac function.

Aurlien et al. identified an *SCN5A* mutation in a patient with idiopathic epilepsy who has died for SUDEP, suggesting that ion channel mutations co‐expressed in the brain and heart can predispose to both epilepsy and arrhythmias.^61^ Moreover, a pathogenic variant in *SCN5A* (p.W1095X, c.3284G>A) has been identified in a family with Brugada syndrome and epilepsy, suggesting that it underlies both cardiac and brain involvement, probably at different developmental ages in the same individual.^62^

A retrospective analysis of 86 autopsies of SUDEP cases analyzed the three main genes associated with LQTS (*SCN5A*, *KCNH2,* and *KCNQ1*) and identified six genetic mutations in *KCNH2* and *SCN5A* genes previously reported in LQTS patients.^63^ In 2015, De Llano et al. identified a *KCNQ1* mutation in a family suffering from epilepsy and LQTS, suggesting that *KCNQ1* genetic variations may confer susceptibility for recurrent seizure activity that increases the risk of sudden death^64^ (Table 2).

**Table 2:** Main genes associated with SUDEP according to different studies.

Genes	*N* articles	Glasscock_2014	Bagnall_2015	Goldman_2016	Devinsky_2016	Bagnall_2017	Thom_2018	Li_2019
Heart genes								
*KCNQ1*	**7**							
*SCN5A*	**7**							
*KCNH2*	**6**							
*RYR2*	**5**							
*HCN4*	**3**							
*KCNQ2*	**2**							
*NOS1AP*	**2**							
*SENP2*	**1**							
*LDB3*	**1**							
*DSC2*	**1**							
*KCNE1*	**1**							
Brain genes								
*SCN1A*	**7**							
*SCN8A*	**7**							
*KCNA1*	**5**							
*HCN2*	**5**							
*SCN2A*	**4**							
*PRRT2*	**3**							
*DEPDC5*	**3**							
*CSTB*	**2**							
*TSC1, TSC2*	**2**							
*SCN1B*	**1**							
*KCNT1*	**1**							
Chromosomal disorders								
*Dup15q11*	**1**							
*5q14.3 Del*	**1**							

The association between SUDEP and the specific genes in the described studies is identified by grey shades

Glasscock (2014),^65^ Bagnall (2016),^66^ Goldman (2016),^38^ Devinsky (2016),^1^ Bagnall (2017),^37^ Thom (2018),^36^ Li (2019).^58^

Until then, few genes had been analyzed in molecular autopsies but with the advent of the NGS era, new candidate genes can now be interrogated (Table 3). Initially, custom resequencing panels including arrhythmogenic cardiac genes and epilepsy genes were the best approach for the genetic screening of SUDEP cases. Coll et al. identified, in 13 and 20 SUDEP and epilepsy patients respectively, variants with complete segregation analysis in *SCN1A*, *FBN1*, *HCN1*, *SCN4A*, *EFHC1*, *CDKL5*, *CNTNAP2*, *GRIN2A,* and *ADGRV1* genes and one copy number variant in the *KCNQ1* gene using a custom resequencing panel.^35^, ^67^ Bagnall et al. performed the largest genetic study of SUDEP using WES in 61 SUDEP cases, identifying mutations known to cause LQTS in 7% of cases and candidate variants in genes potentially predisposing to malignant cardiac arrhythmia in a further 15% of the cases.^66^

**Table 3:** New candidate genes to be associated with SUDEP using the NGS approach.

Custom panel (*n* = 14)	WES (*n* = 18)	WES (*n* = 14)	WES (*n* = 61)	Custom panel (*n* = 9)	Custom panel (*n* = 20)
Coll (2015)	Leu (2015)	Narula (2015)	Bagnall (2016)	Hata (2017)	Coll (2017)
Heart genes					
*BN1, HCN1, SCN4A*	*CACNB2*	*TTN, CACNA1C, JPH2, MYH7, VCL*	*ANK2, AKAP9*	*LDB3, MYBPC3, MYH6, DSP, DSG2*	
Brain genes					
*EFHC1, CACNA1A, SCN10A, SCN11A*	*LGI1*		*GABRB3, PAFAH1B1, CHRN4, PCDH19, SPTAN1*		*CDKL5, CNTNAP2, GRIN2A, ADGRV1*
Other genes					
	*PIK3D2AM LGI1, SMC4, COL6A3, TIE1*				

Bold values are used to identify the genes (cardiac, brain, other genes) investigated in the single studies.

Coll 2015,^35^ Leu 2015,^68^ Narula 2015,^69^ Bagnall 2016,^66^ Hata 2017,^70^ Coll 2017.^67^ WES, whole‐exome sequencing; N, number of cases analyzed.

A recent review suggests different genes potentially contributing to SUDEP: (i) sodium and potassium ion channels subunits were the most frequently reported variants discovered by molecular autopsy, (ii) the *DEPDC5* gene was the second‐highest ranked variant, (iii) the majority of DS patients carried a pathogenic variant in the *SCN1A* gene, and (iv) CNVs in chromosome 15 were associated with autism and a high frequency of epilepsy and SUDEP.^6^ Nevertheless, the functional effect of most of the variants identified by NGS cannot be determined and their pathogenicity remains undefined. Even if the MORTEMUS study identified respiratory dysfunction preceding SUDEP, this research did not find any rare variant in five genes potentially involved in congenital central hypoventilation syndrome.^66^, ^71^

### Closed‐loop recording

Cardiac monitoring is recommended in high‐risk epileptic individuals to supervise and treat potential cardiac arrhythmias and prevent SUDEP or other syncope events.^72^ Implantable loop recorders (ILR) are event recorders that continually analyze the ECG and retain information about significant arrhythmias. They can consistently document a correlation between the presence of symptoms and arrhythmias, as well as exclude a causative role of heart rhythm disturbances in determining syncope or palpitations when they occur without any arrhythmia.^73^

Long‐term studies using ILR for up to 2 years yielded conflicting results, which may be explained by the small sample sizes as well as differences in the selection criteria. Moreover, no efforts were made to discriminate between seizure‐related and non–seizure‐related causes of asystole, including reflex syncope.^74^, ^75^ Recently, a long‐term study on a large cohort of subjects with epilepsy did not find any clinically relevant arrhythmias in individuals with refractory focal epilepsy. Nevertheless, there are no clear guidelines on the use of ILR in preventing death in high‐risk epilepsy patients.^76^

### Therapy‐related changes

Several ASMs can cause abnormalities in the cardiac conduction system, principally in predisposed patients. ASMs acting as sodium channel blockers, for example, carbamazepine (CBZ) or phenytoin (PHT), can give rise to sinus bradycardia, sinus arrest, and AV block, whereas lacosamide has shown a tendency to induce AV block.^7^, ^77^ Also, the QT interval can be increased by retigabine and CBZ, whereas primidone (desoxyphenobarbital) can shorten it. Phenobarbital, lamotrigine (LTG), and PHT can inhibit the IKr and a possible role in SUDEP has been suggested.^78^, ^79^ As GTCS are the single most important risk factor for SUDEP, effective ASMs are crucial in prevention strategies. Although numerous case–control studies suggested ASMs as a strong SUDEP risk factor, particularly when given as polytherapy or when more than two changes in ASM occur per year, literature data have not confirmed this hypothesis^4^. In 1998, CBZ was first associated with a possible risk factor of SUDEP in idiopathic generalized epilepsy, but later studies contradict this theory.^80^ Furthermore, larger studies support the idea that neither CBZ nor any ASM (in mono or polytherapy) is related to SUDEP risk.^81^, ^82^ Also, data from a meta‐analysis by Ryvlin et al. evidence that the use of polytherapy is not associated with an increased risk for SUDEP.^82^, ^83^ A specific attention has been recently focused on the cardiac effects of LTG, which has been associated with an increased incidence of serious adverse cardiac events,^84^ probably related to the inhibition of the cardiac IKr current leading to fatal arrhythmia in genetically predisposed individuals.^78^ Although a combined analysis of 289 SUDEP cases and a case–control study identified a significantly higher risk of SUDEP in treatment with LTG,^85^, ^86^ large‐scale analyses concluded that, after correcting for the frequency of the GTCSs, the use of LTG does not increase the risk of SUDEP.^81^ A recent case report revealed a mutation in the *SCN9A* gene (highly expressed in the brain) causing epilepsy and electrocardiographic Brugada syndrome pattern under LTG administration,^87^ reinforcing the theory that ASM acting on ion channels could increase the risk of arrhythmia and SUDEP in patients with a genetic predisposition. Advances in detecting mutations that may predispose patients to serious cardiac arrhythmias will hopefully enable clinicians to improve safety in the treatment of people with epilepsy and reduce the risk of SUDEP.

## Research Perspectives

Recently, the research has been focused on the genetic architecture of SUDEP patients and the connection with the cause of death has been increased with the advent of the new sequencing technologies. This led to the identification of novel genes associated with SUDEP and other candidates to be associated with this condition. Moreover, the better characterization of the function of SUDEP‐associated genes and their encoded proteins contributed to improving the knowledge of ion channel structure and activity.

Further investigation is needed to elucidate the role of genetic predisposition in the risk of SUDEP. The main issue in the genetic analysis of SUDEP cases (usually post‐mortem) was the availability of high‐quality DNA for NGS analysis. Therefore, it is crucial to increase the awareness of the importance of collecting blood samples in the framework of SUDEP individuals to identify additional causes over autopsy alone.^37^ In most cases, high‐quality DNA from blood samples necessary for NGS did not exist and only formalin‐fixed paraffin‐embedded (FFPE) from surgical tissue specimens were accessible, which made molecular analyses impossible due to the poor quality and high degradation of the DNA. Recently, new high throughput protocols offer successful DNA sequencing from FFPE samples.^88^ Even though this FFPE DNA is not the best option for molecular autopsy, it may offer an alternative source of DNA when postmortem blood or high‐quality extracted DNA is not available. Additionally, a genetic study by NGS from epileptogenic tissue coming from FFPE will allow defining if the identified variants are somatic or germline, and the distribution of the genetic variants throughout the brain, heart, or other tissues. Consequently, the prospective collection of DNA from these tissues could also increase the range and depth of investigations.

Some researches focus on the genetic cause of SUDEP as an extensive polygenic contribution. The WES approach was used to identify in SUDEP cases a higher burden of deleterious genetic variants, with a higher cumulative deleteriousness score, compared to the burden in people with epilepsy who had not succumbed to SUDEP and compared to the burden in people without epilepsy.^68^ As in other disorders, the main limitation in the genetics of SUDEP was the lack of functional studies of a large part of the variants detected by NGS.

SUDEP shared many research limitations with other rare diseases, such as logistical difficulties including incomplete (or absent) autopsies, lack of adequate postmortem DNA samples or appropriate consent, single cases, or small series. Additionally, available data often do not include deep phenotyping or DNA from family members to establish good segregation studies, making novel variant discovery laborious.^6^ Some of these limitations could be partially solved with the extended use of international biorepositories by the scientific community to advance SUDEP research.^36^

## Conclusion

This work shows that epilepsy is associated with a high rate of cardiac comorbidities, and that, during the peri‐ictal period, patients can show changes in the HR, HRV, and ECG abnormalities and develop arrhythmias and TTS. The analysis of registered SUDEP events, together with the use of experimental models and the expansion of the genetic background of SUDEP helped to identify the contribution of cardiac dysfunction in the pathogenesis of SUDEP, which is not completely understood. The foundation of teamwork involving different professional areas such as pathology, neurology, epileptology, cardiology, and genetics, is a fundamental step to expand the comprehension of cardiac involvement in epilepsy and SUDEP and, therefore, to improve the strategies for its prevention.

## Conflict of Interest

P.S. has received speaker fees and participated at advisory boards for BioMarin, Zogenix, GW Pharmaceuticals, and has received research funding from ENECTA BV, GW Pharmaceuticals, Kolfarma Srl., Eisai. The other authors do not report any conflict of interest.

## Authors’ Contributions

GC, AO, MC, and RB contributed to drafting the manuscript, which was critically revised by PS and PP. All co‐authors have seen and approved the submitted version of the paper and accept responsibility for its content.
